# 

**Published:** 1834-07-01

**Authors:** 


### METEOROLOGICAL REGISTER,

### NOTICES.

*We have received* Dr. KAY *on Asphyxia,* Dr. WILSON PHILIP *on Sleep and Death,* Dr. HENDERSON'S *Translation of Raspail,* Mr. HEMING'S *Translation of Mad. Boivin, three Parts of a Cyclopædia of Practical Medicine and Surgery, published at Philadelphia, the Dublin Journal of Medical and Chemical Science, and several Fasciculi of* Dr. QUAIN'S *Anatomical Plates: we shall review them in our next.*

*We also beg to acknowledge the receipt of a "Sketch of the Medical Topography of the Hundred of Penwith, comprising the District of the Land's End, in Cornwall," by* Dr. FORBES. *This interesting Paper forms a part of the second Volume of the Transactions of the Provincial Medical Association, which we have reviewed in our present Number.*

*We regret that, owing to an unforeseen disappointment, we have not been able to review* Dr. BOW'S *Work on Inflammation in this Number; but we shall not fail to do so in our next.*

*Considering it sufficient to give an account of Books actually published, we think it unnecessary to tantalize our readers with probable or possible Books; and we therefore abstain from giving what is called "Literary Intelligence." Those advertisements of Books about to be published can appear, however, in our Advertising Sheet.*

*Contributors are requested to favour us with their Communications before the* 16*th of the month preceding publication.*

*We have not inserted* Dr. WILLIAMS'S *Letter, as it seems to have been sent as a circular to all the Medical Journals: it was printed in the Lancet a few weeks since.*

Mr. HOWSHIP'S *Paper on the Æstrus Humanus has been received, and will appear in No. V.*

### INDEX TO VOL. II.

Page.
ABSCESS in the groin, from swallowing a pin, case of . . 403
of the thigh, case of deep-seated . . . 459
Absorbents, function of the . . . . 299
Abuse of spirituous liquors, Mr. Marshall on the . . 281
Acetate of lead should be prescribed with vinegar . . 40
Aconite, homœopathic formula for administering . . 400
Adams's Translation of Paulus Ægineta, reviewed . 6 and 9
Advice, every one gets the best he can afford . . . 493
Alphabet of Medical Botany, by Professor Rennie } reviewed 50 *et seq.*
Botany }
Ammoniacum, its origin . . . . 44
Amputation of the thigh, cases of . . . 412, 414, 415
Anatomical Plates, by Jonas Quain, M.D., reviewed . 146
Anatomy, Introduction to the Study of, by James Paxton, reviewed . 148
general . . . . 347
of the Eye, Dalrymple on the, reviewed . . 340 *et seq.*
and Physiology of the Liver, Mr. Kiernan on the, reviewed 288 *et seq.*
Animal Magnetism, Mr. Colquhoun on, reviewed . 135 *et seq.*
Dr. Pritchard's observations on . . 137
magnetism, theory of . . . . 484
temperature . . . . . 373
Anchylosis, Sir B. C. Brodie on . . . . 258
Apparent direction of eyes in a portrait . . . 212
Aretæus knew the division of the nerves into sensatory and motory . 66
editions of . . . . . 142
quoted . . . . . . 365
Armstrong's Lectures, reviewed . . . 1 *et seq.*
Armstrong erroneously supposed the ancients ignorant of anatomy . 2
Arsenic, Dr. Murray on the tests for . . . 287
Ashburner on Dentition, reviewed . . . . 147
Atkinson's Medical Bibliography, reviewed . . 141
Auscultation obstetric . . . . . 83, 274
Bardsley on the efficacy of strychnine in paralysis . 341
Bathing neglected in this country . . . . 13
Bell Sir C., Mr. Mayo on the discoveries of . . 450
Belladonna, its efficacy in strangulated hernia . . . 465
Billard on the Diseases of New-born Infants, reviewed . 111 *et seq.*
Births in Paris during the years 1831 and 1832 . . . 214
Blood, analysis of in a case of cholera . . . 107
Bloodletting as a diagnostic mark of disease . . . 325
Bloxam Mr. Wm., his Cyclopædia of Practical Surgery, reviewed . 399
Blundell, Dr. on the Principles and Practice of Obstetricy, reviewed 308 *et seq.*
Boarding-schools for girls, their bad effects on health . . 389
Botany, Professor Rennie's mistakes in . . . 50 *et seq.*
Brain the sole organ of the will, according to Le Gallois . . 33
Bridgewater Treatise, Dr. Prout's, reviewed . . 267 *et seq.*
Brodie on the Diseases of the Joints, reviewed . 241 *et seq.*
Bronchocele, observations on the treatment of, by James Reid, esq. . 425
cases of . . . . 439 *et seq.*
Calculous diseases said to be rare among sailors . . 326
Caries of the spine . . . . . . 259
Carswell, Dr. Illustrations of the Elementary forms of Disease by, reviewed
131 *et seq.*, 397 *et seq.*
Catarrhal and Catarrho-rheumatic ophthalmia, observations on, by Frederick
Tyrrell, esq. . . . . . . 416
Celsus quoted . . . . . . 3, 55

Chlorine, inhalation of . . . . . 41
its action on metallic iodides . . . 491
Cholera, the Croonian Lectures on, by Dr. Roupell, reviewed . 97 *et seq.*
malignant, a disease of modern origin . . 97
is it contagious? . . . . . . 98
earliest cases of in London . . . 99
bleeding in . . . . . 100
emetics in . . . . . 103
salines in . . . . . 104
stimulants in . . . . . *ib.*
purgatives in . . . . . 105
narcotics in . . . . . *ib.*
malignant, its resemblance to asthenic fever . 107
Chomel's Lectures on Typhus Fever, reviewed . . 343 *et seq.*
Chorea, sancti Viti, case of . . . . . 196
Circular and flap operations, comparison of the . . 411
Dr. Tolefree on the . . 452
Club-foot cured by division of the tendo-Achillis . . 466
Codeine, therapeutic effects of . . . . 206
Colchicum in erysipelas . . . . . . 183
its use in inflammation of the synovial membrane . . 248
College of physicians . . . . 234
a well-regulated body . . . 395
Colquhoun on Animal Magnetism, reviewed . . 135 *et seq.*
Combe, Dr. on the Preservation of Health, reviewed . 388 *et seq.*
Compilation, the difficulty of . . . . 54
Condylomata cured by creosote . . . . 453
Congestion of the brain in new-born infants . . 114
Congestive fever, Dr. Armstrong's account of . . . 10
Conolly, Dr. his life of Dr. Darwall . . . 367
Constipation, case of, successfully treated by the introduction of air into the bowels . . . . . . . 203
Constitution of a patient to be inquired into . . . 328
Consumption Curable, by Dr. Ramadge, reviewed . . 320 *et seq.*
Copaiba, method of ascertaining its purity . . . 45
Cornea, on the structure of the covering of the . . 215
Counterblaste to Tobacco, King James's . . . 479
Cours Théorique et Pratique d'Accouchemens; par J. Capuron, reviewed 273 *et seq.*
Crotchet the, detestable . . . . . 279
Crowing inspiration of children, Dr. Hugh Ley, on the . 205
Curtis, on the Preservation of Sight, reviewed . . . 144
Cyclopædia of Practical Medicine, Part XX., reviewed 78 *et seq.*
———— of Practical Surgery, reviewed . . . 399
Dalrymple, on the Anatomy of the Eye, reviewed . 340 *et seq.*
Davies, Dr. Henry, Cases extracted from the Note-book of . 179, 402
Dead bone, absorption of, in necrosis . . . 301
Deafness, case of . . . . . . 21
chronic, method of ascertaining its intensity . 18
irritative and torpid, nervous . . . 25
pathology of, hitherto neglected . . . 17
Death of new-born infants from anormal states of the umbilical cord 462
Deaths in Paris in 1831 and 1832 . . . 214
Demonstration of the Nerves, by Joseph Swan; Part IV., reviewed 134 *et seq.*
Dental Guide, the Parents', by William Imrie, reviewed . . 150
Dentition, Dr. Ashburner on, reviewed . . . 147
Diagnosis, Dr. M. Hall on the Principles of, reviewed . 325 *et seq.*
Diagnosis of cardiac diseases by the stethoscope is still in an embryo state 471
Digestion, Dr. Prout on the function of . . . 270
Digitalis; in what dose should it be given . . . 454
cases of poisoning by . . . *ib.*

Diseased joint, post-mortem examination of . . . . 302
Diseases, origin of . . . . . . 379
of children, Dr. Walker on the . . . 364
Dislocation of the shoulder . . . . 363
Dispensaries, self-supporting . . . . . 494
Dissertations on Points of Practical Medicine and Surgery, by Dr. Richter, reviewed . . . . . 331 *et seq.*
Dissection of the nerves, observations on the . . . 135
Dowson's Introduction to the Study and Practice of Medicine, reviewed 151
Dropsy, Dr. Armstrong on the treatment of . . . 204
Druggists' practice legal, according to Gray . . 492
Drugs, state of, in London . . . . . 481
Dyspepsia biliosa, efficacy of emetics in . . . . 45
case of . . . . . . *ib.*
Ear, chronic diseases of, divided by Kramer into three classes . 20
Earl of Bridgewater left an eccentric legacy . . . 268
Eccles on the Ulcerative Process, reviewed . . . . 137
Ecstasis, remarkable case of . . . . . 78
Edinburgh graduates . . . . . . 140
Medical and Surgical Journal, Nos. CXVIII. and CXIX., reviewed 280 *et seq.*
Elongation of the limb in incipient disease of the hip-joint . 307
Embryotomy, the most knotty point in medical ethics . . 311
Empyema cured by operation . . . . . 191
Encephaloid structure, case of conversion of the right lung into an . 468
Encysted omental dropsy, case of . . . . 404
Equilibrium, sense of, Mr. Herbert Mayo on the . . 405
Erysipelas, cases of, treated by colchicum . . . 183
Erysipelatous inflammation of the meatus auditorius . . 21
Essay on the Physiology of the Iris, by Mr. Walker, reviewed . 150
Examination into the Causes of the declining Reputation of the Medical Faculty of the University of Edinburgh, reviewed . 139 *et seq.*
Experiments for determining the sentient qualities of nerves not very satisfactory, 91
Eustachian tube; Dr. Kramer's method of catheterizing . 19
tube and tympanum, diseases of . . . 23
tubes, case of obstruction of . . . 24
tube, adhesion of its sides . . . . 25
Fairy-rings . . . . . . . 215
Fever protracted, arising from the state of the liver . . 125
Fevers, Paulus Ægineta on . . . . . 73
Flood, Dr. Valentine, his Anatomy of Hernia, reviewed . 399
Fœtus, pathology of the . . . . . 473
of the whale . . . . . . 231
Foramen of Soemmering . . . . . 340
Fordyce on fever, quoted . . . . . 108
Forestus, quoted . . . . . . 318
Fox, Douglas, on the Signs, Disorders, and Management of Pregnancy, reviewed 401
Foxglove stimulates the genitals . . . . . 48
its inutility in hydrocephalus . . . . 48
Fractures of the leg, use of gypsum in the treatment of . 331
Fumigating Baths, Observations on, by Jonathan Green, reviewed . 146
Fungus hæmatodes of the bones, case of . . . . 263
Galen, his views of the nervous system . . . . 66
Gardener's Dictionary, by Philip Miller, reviewed . . 390
Gastric fever, Mr. Poole on a . . . . . 281
Giddiness leads to nausea and vomiting . . . 480
Graves, Mr. F. his Hortus Medicus, reviewed . . 385 *et seq.*
Green on Fumigating and other Baths, reviewed . . 146
Gypsum, use of, in treating fractures of the leg . . . 331

Hæmaturia, astringents useful in . . . 41
Hæmorrhoids, Dr. A. T. Thomson's observations on . . 40
Hall, Dr. M., on the Reflex Function of the Medulla Oblongata, and the Medulla Spinalis, reviewed . . . 25 *et seq.*
on the Principles of Diagnosis, reviewed . 32 *et seq.*
Heart, functional derangement of the, remarkable case of . 126
foreign body found in the . . . . 363
Dr. Spittal, on the diseases of the . . . . 285
its temperaments, according to Paulus Ægineta . . 71
Dr. Armstrong, on chronic disorders of the . . 471
Hemiplegia, table of cases of, treated with strychnine , . 355
Hemorrhage from leech bites, method of stopping . . 14
Hepatic veins . . . . . . 289
Hernia, the Anatomy and Surgery of Inguinal and Femoral, by E. W. Tuson, reviewed . . . . . 145
Hernia, Anatomy of, by Dr. Valentine Flood, reviewed . . 399
Hernia strangulated, case of, with adhesions to the sac, by Mr. Valentine 448
Hidrotic fever . . . . . . 314
Hippocrates quoted . . . . . 65
on erysipelas of the parts about the throat . . 75
Hippomaneæ . . . . . . 226
Hohl, Dr., Treatise on Obstetric Exploration by, reviewed . 83 *et seq.*
Holland, Dr. G. Calvert, Inquiry into the Principles and Practice of Medicine, reviewed . . . 370 *et seq.*
Homœopathy . . . . . 228
Homœopathic notation . . . . . . 401
Hooker's Journal of Botany, Part I., reviewed . . . 149
Hope's, Dr., Illustrations of Morbid Anatomy, reviewed . 367 *et seq.*
Horse-shoes . . . . . . . 218
Horse, symptoms of pain in the . . . . . 489
Hortus Medicus, by Mr. Graves and Dr. Morris, reviewed . 385 *et seq.*
Hospital physicians should improve the practice of physic . . 343
student, the . . . . . . 391
Hospitals, advantages of small . . . . 490
Howship, Mr., case of meningitis, by . . . 446
Hunter, John . . . . . . 489
Hutchison, Mr. Copland, his great improvement in the treatment of erysipelas . . . . . . 461
Hydatids, case of, by Dr. Craigie . . . . 284
uterine case of . . . . . 363
Hydrocyanic acid, test for . . . . . 217
Hydrophobia, case of, by Mr. Grindrod . . . 360
Hydrostatic test, Mr. Jennings on the . . . 365
Hypertrophy, seats of . . . . . 368
of the mammæ, case of . . . 477
Hypochondriasis, case of . . . . 189
in medical pupils . . . 190
Illustrations of the Elementary Forms of Disease, by Robert Carswell, M.D., Fasc. I.—IV., reviewed . . . 131 *et seq.*
Fasc. V., reviewed . . . . 397
of Morbid Anatomy, by Dr. Hope, reviewed . 367 *et seq.*
Imrie's Parents' Dental Guide, reviewed . . . 150
Inflammation of the abdomen . . . . 327
of synovial membrane, symptoms of . . 246
treatment of . . 247
Inflammatory softening of the cellular tissue . . . 397
Inhalation of Iodine and Chlorine in Phthisis, Treatise on the, by Sir Charles Scudamore, reviewed . . . . 127 *et seq.*
Sir C. Scudamore's directions for . . . 128
Dr. Ramadge on . . . . 321
Inosculation of the veins of the liver . . . . 291
Inquiry into the Principles and Practice of Medicine, by Dr. G. Calvert Holland, reviewed . . . . 370 *et seq.*

Insanity, Dr. Smith on . . . . . . 284
Interstitial absorption of bones . . . . 337
Introduction to the Study and Practice of Medicine, by John Dowson, M.D. reviewed . . . . . . 151
Intus-susception, on the sanative process of nature in . . 337
Iodine, Dr. Lugol's formulæ for the administration of . . 47
Ioduret of iron . . . . . . . 82
method of preparing the . . . *ib.*
Jacob, Dr., his membrane . . . . . 342
Jalapine, its characters . . . . . 46
Joints, Wickham on the Diseases of the, reviewed . . 296 *et seq.*
Journal of Botany, Part I., by Dr. Hooker, reviewed . . 149
Key on the Ulcerative Process in Joints, reviewed . . 296 *et seq.*
Kidney, tuberculous affection of the, case of . . . 361
Kiernan on the Anatomy and Physiology of the Liver, reviewed 288 *et seq.*
Knee-joint, method of puncturing . . . . 249
Kramer on Chronic Deafness, reviewed . . . 17 *et seq.*
Labour tedious, from adhesions in the vagina, case of, by Dr. H. Davies 181
Laryngeal nerves, effect of the division of the superior . . 28
Laryngitis, case of, cured by inhalation . . . 129
Leech-bites, Dr. Thomson's method of stopping the bleeding from . 40
Leeches preferred to cupping by Dr. Armstrong . . 13
Lecturer, qualifications of a good . . . . 1
Lectures, their advantages and disadvantages . . . 308
Licentiate of the College of Physicians, his pamphlet on Medical Education, reviewed . . . . . . 391 *et seq.*
Lithotomy by the rectum, case of, by Mr. J. Dawson . . 357
in Italy . . . . . 196
Liver, Mr. Kiernan on the functions of the . . . 295
its temperaments, according to Paulus Ægineta . . 73
case of tumour in the region of the, by Dr. Macnish . . 286
Lucretius quoted . . . . . 485
Maceration of different textures of the body in water . . 286
Macilwain, Mr., letter from . . . . 207
Madhouses, regulation of . . . . . 208
Malignant disease of the joints, case of . . . 262
Mammæ, case of hypertrophy of the . . . 477
Marasmus, case of . . . . . . . 402
with omental tumour, case of . . . 403
Marriages and births, their relative proportion . . . 81
Mayo, Mr. Herbert, on the sense of equilibrium, and on its disturbance 405
on the circular and flap operations . . 411
on Sir C. Bell's discoveries . . 450
Mead, Dr., quoted . . . . . . 396
Measles mentioned by the Arabian writers . . . 76
Meatus auditorius imperforate, case of . . . 95
Medica Sacra, reviewed . . . . . 396
Medical Bibliography, by James Atkinson, reviewed . . 141 *et seq.*
reform . . . . . . 232
Medicine, the physiology of the sick man . . . 371
Medicines, on the classification of, by J. Stevenson Bushnan, F.L.S. 152 *et seq.*
Medulla spinalis, its reflex fluction . . . . 26 *et seq.*
Meningitis, case of, by Mr. Howship . . . . 446
Mercury, on the Influence of Minute Doses of, by Dr. A. P. W. Philip, reviewed . . . . . . . 121 *et seq.*
Metamorphosis of plants . . . . . 52
Metastasis of diseases . . . . . 326
Meteorological Register . . . . . . 240, 496
Miller, Philip, his Gardener's Dictionary reviewed . . 390
Morbid formation of bone in the muscles . . . 334
Morris, Dr., his Hortus Medicus reviewed . . 385 *et seq.*
Muscular action, its four modes . . . . 26
sense . . . . . 406

Nerves, a Treatise on Diseases and Injuries of the, by Joseph Swan, reviewed . . . . . . 90 *et seq.*
their painful affections . . . . 90
partially divided . . . . . 94
diseased cases of . . . . . 200
Nervous sympathy generally recognized by physiologists . . 29
Neuralgia, intermittent case of . . . . 92
two cases of, cured by bark . . . 93
New-born Infants, Treatise on the Diseases of, by Dr. Billard, reviewed 111 *et seq.*
Nightmare, Dr. Strahl on, reviewed . . 315 *et seq.*
its symptoms . . . . 315
diagnosis of . . . . . 317
prognosis of . . . . *ib.*
treatment of . . . . 318
Nosology, new Synopsis of, by Dr. Weatherhead, reviewed . 383 *et seq.*
Notices . . . . . . . 240, 496
Oaks, great . . . . . . . 211
Observations on the Preservation of Sight, by John Harrison Curtis, Esq. reviewed . . . . . . 144
on the Present System of Medical Education, reviewed 391 *et seq.*
on the Ulcerative Process, by Mr. Eccles, reviewed 137 *et seq.*
Obstetric Exploration, Treatise on, by Dr. Hohl, reviewed 83 *et seq.*
Obstetricy, the principles and practice of, by Dr. J. Blundell, reviewed 308 *et seq.*
Organs of the voice in birds . . . . . 221
Os uteri, case of the obliteration of the, by Mr. William Taylor . 189
Oxford medical degrees . . . . . 234
Paralysis of one side of the face, case of . . . 190
cases of, cured by strychnine . . 352 *et seq.*
case of, cured by application of the tourniquet . . 188
Paraplegia, table of cases of, treated with strychnine . . 356
Pathological and Surgical Observations on the Diseases of the Joints, by B. C. Brodie, V.P. R.S., reviewed . . 241 *et seq.*
Paulus Ægineta, translation of, by Mr. Adams, (Vol. I.) reviewed 60 *et seq.*
his history and æra . . . . 62
Paxton's Introduction to the study of Anatomy; Vol. II., reviewed 148
Pelvis, differences between the male and female . . . 309
Perineum, its support during labour, unnecessary . . 310
derivation of the word . . . . 315
Peritonitis, chronic, Mr. Edward Thompson on . . 357
Pharmacopœia Homœopathica, by Dr. Quin, reviewed . 399 *et seq.*
Philip, Dr. Wilson, on the Influence of Minute Doses of Mercury, reviewed 121 *et seq.*
Phlegmasia dolens in men, Sir H. Halford on . . 475
Phthisis, Dr. Ramadge's method of curing . : . 321
Physiology applied to the Preservation of Health, by Dr. Combe, reviewed 388 *et seq.*
Piscidia erythrina, or the fish-wood . . . . 230
Plural births . . . . . . 279
Polypus in the meatus auditorius . . . . 21
Post-mortem appearances after an overdose of strychnine . 354
Pregnancy, Capuron on the diagnosis of . . . 273
case of supposed . . . . . 87
Propagation of heat and light below the earth's surface . . 268
Prout, Dr., his Bridgewater Treatise, reviewed . . 267 *et seq.*
Puerperal convulsions . . . . . . 277
Pulmonary consumption, case of, by Dr. William Stroud . 171 *et seq.*
Pulse, its varieties . . . . . . 4
fœtal . . . . . . 84
placental . . . . . . *ib.*
Pulse, its variation during labour . . . 86

Quain's Anatomical Plates reviewed . . . 146
Quin's Pharmacopœia Homœopathica, reviewed . 399 *et seq.*
Ramadge Dr., his "Consumption Curable," reviewed . 320 *et seq.*
Reflex function of the medulla spinalis, not discovered by Dr. M. Hall 29
Reid, James esq. on the treatment of bronchocele . . 425
Relation of the nervous to the vascular system . . 372
Relaxation and descent of uterus and bladder . . . 287
Rennie's Alphabet of Botany, reviewed . . 50 *et seq.*
Magazine of Botany and Gardening, reviewed . 394 *et seq.*
Rheumatism of the elbow-joint . . . . 337
Richmond, Mr., his musical performance . . 483
Richter's Dr. A. L., Dissertations on Points of Practical Medicine and Surgery, reviewed . . . . . 331 *et seq.*
Roupell, Dr., Croonian Lectures on Cholera by, reviewed . 97 *et seq.*
Rupture of a varicose tumour in the vagina during labour . 455
Ruspini's styptic . . . . . . 40
St. Pancras Association, observations on the . . 494
Salivation excited by minute doses of mercury . . 122
Scott, Mr., his method of bandaging . . . . 255
Scrofulous disease of the joints . . . . 256
Scudamore, Sir C., on Inhalation in Phthisis, reviewed . 129 *et seq.*
Secale cornutum, its use . . . . 276
Seeds, Professor Rennie's account of . , . . 53
Self-supporting dispensaries . . . . 494
Shapter, Dr., his Medica Sacra, reviewed . . . 596
Signs, Disorders, and Management of Pregnancy, by Mr. Douglas Fox, reviewed . . . . . . 401
Sloe, its medical virtues . . . . . 39
Small-pox mentioned by the Arabian writers . . 76
Sneezing . . . . . . 456
Soemmering ; his account of the relative dimensions of the male and female pelvis . . . . . . 309
Softening of organs, Dr. Carswell on . . - 78
Solon quoted . . . . . 136
Somnambulism and animal magnetism, Dr. Pritchard on . 78
Sounds inaudible by certain ears . . . . 220
Spark produced during the freezing of water by æther . . 217
Spontaneous evolution, case of . . . . 286
State medicine in Germany . . . . 486
Stays, their absurdity . . . . . 389
Stethoscope, its construction . . . . 81
Stomach, its temperaments, according to Paulus Ægineta . . 70
affections of the, in new-born infants . . 117
case of . . 118
dilated, cases of . . . . 201
Stools, their colour . . . . . 7
resembling mock-turtle soup, produced by calomel . 8
darkened by iron, sulphur, and senna . . . 9
Strahl, upon Nightmare, reviewed . . . 315 *et seq.*
Strangulated hernia, reduction of . . . . 231
efficacy of the extract of belladonna in . 465
Stroud, Dr., his case of pulmonary consumption . . 170
Strychnine, its efficacy in paralysis . . . 351
Sudamina, their occurrence in typhus fever . . . 545
Sulphate of magnesia, Dr. Henry on the . . . 282
zinc, the best emetic in congestive fever . . 11
Surgical anecdote . . . . . 219
Swan, on Diseases of the Nerves, reviewed . . 90 *et seq.*
Swan's Demonstration of the Nerves, Part IV. reviewed . 134
Sympathetic nerve, case of disease of the . . . 457
Synovial membrane, case of inflammation of . . . 244
Tannin, pure . . . . . . 490

Taxodium . . . . . . 221
Temperaments of the brain . . . . . . 64
Tendo-Achillis divided for the cure of club-foot . . 466
Tetanus cured by cupping along the spine . . . 467
Thomson's, Dr. A. T., Elements of Materia Medica(Vol. II.) reviewed, 39 *et seq.*
Tourniquet applied in paralysis . . . . . 188
Trachea of birds . . . . . . 222
Transactions of the Provincial Medical and Surgical Association, Vol. II., reviewed . . . . . 351 *et seq.*
Transfusion, Dr. Blundell's directions for . . . 312
Triple quotidian ague, case of . . . . . 479
Truth, Dr. Blundell's panegyric on . . . . 311
Tubercular disease, curability of . . . . 131
Turnbull, Dr. A., Treatise on Veratria by, reviewed . 55 *et seq.*
— writes not for the profession, but the laity . 57
— Wm., his Medico-chirurgical Report of the Huddersfield Infirmary . . . . . . 280
Turtle, experiment upon, by Dr. M. Hall . . . . 33
Tuson's Anatomy of Hernia, reviewed . . . 145
Twin cases detected by auscultation . . . 88
Typhus fever, Dr. Armstrong's account of the post-mortem appearances in 14
— remarkable case of, related by Rush . . 15
— Chomel's Lectures on, reviewed . . 343 *et seq.*
— symptoms of . . . . . 344
— post-mortem appearances in . . . 346
— causes of . . . . 347
— case of, narrated by Professor Chomel . . . 349
— treatment of . . 350
Tyrrell, Mr. F., on catarrhal and catarrho-rheumatic ophthalmia . 416
Ulcerative Process in Joints, Key on the, reviewed . . 296 *et seq.*
Ulceration of the cartilages, Mr. Wickham's account of . . 305
— of the cartilage of the hip-joint, Mr. Key on . 303
— of the cartilages, Mr. Key's account of the . . 297
— of the articular cartilages . . . 251
— case of . . . *ib.*
— of the eyelid, case of . . . . . 227
Unequal length of the legs, case of . . . . 265
Urine, analysis of, in a case of cholera . . . 187
Valentine, Mr., case of strangulated hernia, by . . . 448
Veins: do they absorb? . . . . 300
Venereal disease, preventive of the . . . . . 16
— treatment of the, in the Hospital of the 72d Regiment, at the Cape of Good Hope . . . . . 459
Venesection in cases of rigid os uteri . . . . 278
Veratria, Treatise on, by Dr. A. Turnbull, reviewed . 55 *et seq.*
Verdigris, case of neuralgia caused by . . . 93
Vindiciæ Medicæ, or a Defence of the College of Physicians, reviewed 394 *et seq.*
Vision, Mr. Mayo on . . . . . . 408
Walker, on the Physiology of the Eye, reviewed . . 150
Watery discharge during pregnancy, case of, by Dr. H. Davies . 179
Weatherhead, Dr., New Synopsis of Nosology, reviewed . 383 *et seq.*
Wellesley Dispensary, Dr. Churchill's report of the . . 281
What can the legislature do for us? . . . . . 492
Whytt quoted . . . . . . 30
Wickham, on Diseases of the Joints, reviewed . 296 *et seq.*
Willow, the weeping . . . . . . 213

END OF VOL. II.

J. and C. Adlard, Printers,
Bartholomew close.

